# Facile Isolation of LCC-Fraction from Organosolv Lignin by Simple Soxhlet Extraction

**DOI:** 10.3390/polym11020225

**Published:** 2019-01-31

**Authors:** Reza Ebrahimi Majdar, Ali Ghasemian, Hossein Resalati, Ahmadreza Saraeian, Claudia Crestini, Heiko Lange

**Affiliations:** 1 Department of Chemical Sciences and Technologies, University of Rome ‘Tor Vergata’, Via della Ricerca Scientifica, 00133 Rome, Italy; rezaebrahimim@gmail.com; 2 Department of Pulp and Paper Science and Technology, Gorgan University of Agricultural Sciences and Natural Resources, Shahid Beheshti, Gorgan, Iran; ali.ghasemian1960@yahoo.com (A.G.); saraeyan@yahoo.com (A.S.); 3 Department of Wood and Paper Science and Industries, Sari University of Agricultural Sciences and Natural Resources, Farah Abad Road, Sari, Iran; hnresalati@yahoo.com; 4 Department of Molecular Sciences and Nanosystems, University of Venice Ca’ Foscari, Via Torino 155, 30170 Venice Mestre, Italy

**Keywords:** organosolv lignin, LCC, fractionation, solvent effects, thermal properties

## Abstract

A new fractionation protocol for wheat straw organosolv lignin was developed on the basis of the dominating H-bonding orientations of its components. Acetone as H-bond accepting aprotic polar solvent and methanol as H-bond donating and accepting protic polar solvent were used in sequence. Obtained fractions were structurally and thermally analysed. The protocol allowed for the generation of purified lignin fractions and the isolation of a novel, yet unobserved lignin carbohydrate complex (LCC) fraction. This LCC fraction was found to contain exclusively phenyl glycosides and γ-esters as LCC motifs.

## 1. Introduction

Abundant non-fossil-based but renewable polyphenolic resources continue to have problems in benefitting from the growing trends of sustainability in general, and substitution of non-sustainable “traditional” active ingredients in every-day consumer products in particular [1,2,3]. Especially lignin seems to continuously suffer from intrinsic peculiarities ranging from differences stemming from natural origins to issues emerging during industrially feasible isolation processes, despite enormous amounts of research papers and patent literature targeting the field in general [4]. Tenacious impurities that cannot be easily removed eventually impede progress as well.

With the aim of tailoring lignins for specific valorisations, lignin-inherent functional groups and molecular weight characteristics are often targeted. Chemical, physical and or physical-chemical separations are used to induce prevalences that do not occur naturally and match the application needs. In terms of molecular weights, a rather obvious and simple way to arrive at tailored lignins is lignin fractionation. Fractionation can be conveniently used to divide out structural features and/or different molecular weight characteristics. Fractionation eventually also allows for identification of non-lignin extractions and impurities. Since fractionation is thus a powerful tool for obtaining less polydisperse and/or cleaner lignin fractions, a huge amount of knowledge has been acquired and reported in this field, using one or more of the following techniques, which can here only be highlighted by exemplary studies, as indicated in the following: the idea of fractionating lignins is not new; it has been investigated on various samples in the 1980s [5], after initial attempts in the early 1950s [6]. Various strategies are discussed in the literature: (i) sequential precipitation out of alkaline solutions [7]; (ii) fractional precipitation of re-dissolved kraft lignin and a wheat straw lignin in a gradually changed binary solvent system [8,9]; (iii) (sequential) extractions using different solvents [7,10,11,12,13,14,15,16]; or (iv) just water [17] followed by adsorption; (v) fractionation by ultrafiltration of black liquor using ceramic membranes [18,19,20]; and (vi) thermal fractionation techniques [21].

Lignin carbohydrate complexes (LCCs) are elusive components of the wood cell, postulated for more than 100 years [22,23]. Several different methodologies have been used in attempts to demonstrate their existence, but final hard proofs are still missing. Nevertheless, hints to their existence exist in the literature, among others, in the form of cross-peaks in bidimensional nuclear magnetic resonance (NMR) analyses. Five principal structural motifs are discussed for LCCs, and are consequently looked for in connection with LCC-detection: phenyl glycosides (**PG**), benzyl ethers (**BE**), γ-esters (**GE**), ferulate/coumarate esters (**FE**/**CE**) and acetal/hemiacetal linkages (**AL**/**HL**) [23,24]. When ruling out other species that could potentially create cross-peaks at positions seen typical for LCCs, direct spectroscopic evidence of their presence in the form of **PG**, **BE** and **GE** have been reported for wheat straw organosolv lignins in the recent past [13,25,26].

The issue of isolating clean LCC fractions for fundamental understanding of wood structure and pulping chemistry is strictly interconnected with the development of fractionation processes able to produce consistent fractions with specific homogeneous characteristics. More specifically, besides the obvious basic differences in molecular weight distribution and phenolic group content, it is of pivotal importance to develop a fractionation methodology able to separate fractions that would differ also in H-bonding characteristics and affinities, which eventually might influence tertiary structure of lignin oligomers and polymers in solutions of different H-bonding characteristics. From this perspective, the development of a new fractionation process allows in principle to tune the overall chemical nature and behaviour of the fractions irrespective of the specific molecular weight.

**Figure 1:**
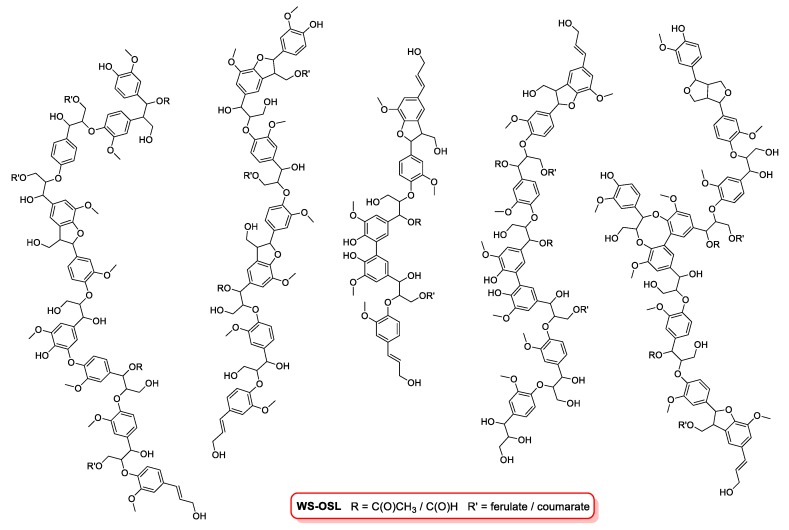
Structural features of organosolv lignins **WS-OSL**.

In this study, our efforts were focused on the fractionation of a wheat straw-derived organosolv lignin (**WS-OSL**) produced via the CIMV organosolv biorefinery process [27], which has been used as a model for lignins stemming from modern organosolv-biorefinery processes of renewable lignocellulosic biomass in various studies before [28,29,30,31,32,33]. Structural features are shown in Figure 1. As indicated in the structural representation, some of the hydroxyl groups in the **WS-OSL** under study are esterified in the form of formates or acetates as reminiscence to the CIMV isolation process; a previous study estimated the amount of esterified OH-groups to be approximately 10% of total aliphatic and aromatic OH-group content [9].

Since the **WS-OSL** under study was known to contain certain carbohydrate impurities, we were especially interested in delineating whether it was possible to remove these carbohydrates impurities in an H-bonding-based scalable fractionation approach.

**WS-OSL** was hence fractionated on the basis of exhaustive extractions using a simple but scalable Soxhlet set-up. Two polar solvents were used: acetone as H-bond accepting aprotic polar component, and methanol as protic polar solvent capable of acting as donor and acceptor in H-bonding. Solvents were applied solely and in sequence, but never in the form of binary systems, with the sequences not necessarily following the order suggested by taking into account polarity in connection with Hanson solubility parameters [34]. Resulting fractions were structurally analysed and studied regarding their glass transition temperatures as function of revealed structural features.

## 2. Materials and Methods

### 2.1. General

Wheat straw organosolv lignin **WS-OSL** was produced via the Biolignin^TM^ process by CIMV (Compagnie Industrielle de la Matière Végétale), Levallois Perret, France [27]. Solvents in appropriate grades were purchased from Sigma Aldrich/Merck KGaA (Darmstadt, Germany) and Carlo Erba (Milan, Italy), and used as received if not stated otherwise.

### 2.2. Soxhlet Fractionation of **WS-OSL**

Typically, 10 g of **WS-OSL** or already derived fractions thereof were placed in a cellulose thimble inside a Soxhlet extractor. The solvent chosen for the respective extraction step, typically 125 mL, was placed in a round bottom flask that was heated by an oil bath. The solvent was brought to reflux and liquid solid extractions were continued until the solvent exiting at the end of an extraction cycle was colourless. Lignin fractions were isolated by drying the thimble and removing the solvent from the liquid fraction in vacuo. Final drying was achieved by placing the samples in a vacuum oven at 40 °C.

### 2.3. ^31^P NMR Analysis

In general, a procedure similar to the one originally published and previously applied was used [34,35]. Approximately 30 mg of the lignin were accurately weighed in a volumetric flask and suspended in 400 μL of a solvent mixture of pyridine and deuterated chloroform (CDCl_3_) (1.6:1 *v*/*v*) the above prepared solvent solution. One hundred microliters of the internal standard solution, i.e., cholesterol at a concentration of 0.1 M in the aforementioned NMR solvent mixture, were added. Fifty milligrams of Cr(III) acetyl acetonate were added as relaxation agent to this solution, followed by 100 μL of 2-chloro-4,4,5,5-tetramethyl-1,3,2-dioxa-phospholane (Cl-TMDP). After stirring for 120 min at ambient temperature, ^31^P NMR spectra were recorded on a Bruker 400 MHz NMR spectrometer (Billerica, MA, USA) controlled by TopSpin 2.1 software, with the probe temperature set to 20 °C. The Bruker sequence zgig in qsim acquisition mode was used with: NS = 64; TD = 32768; SW = 60.0000 ppm; O1 = 42,510.24 Hz; O2 = 3290.40 Hz; D1 = 10 s; acquisition time = 963.4586 ms; and P1 = 6.20 µs. NMR data were processed with MestreNova (Version 8.1.1, Mestrelab Research, Santiago de Compostela, Spain). Drift correction and zero-filling were performed prior to Fourier transform. Chemical shifts were expressed in parts per million (ppm) from 85% phosphoric acid (H_3_PO_4_) as an external reference; all chemical shifts reported were relative to the reaction product of water with Cl-TMDP, which gives a sharp signal in pyridine/CDCl_3_ at 132.2 ppm. The maximum standard deviation of the reported data was 0.02 mmol g^−1^, while the maximum standard error was 0.01 mmol g^−1^ [34,36].

### 2.4. ^1^H−^13^C HSQC Measurements

Samples of around 50 mg were dissolved in 600 μL DMSO-*d*_6_ (providing NMR sample solutions with concentrations of around 83 mg/mL); chromium acetyl acetonate was added as spin-relaxing agent at a final concentration of ca. 1.5–1.75 mg/mL. Heteronuclear single quantum coherence (HSQC) spectra were recorded at 27 °C on a Bruker 400 MHz instrument (Billerica, MA, USA) equipped with TopSpin 2.1 software. The Bruker hsqcetgp pulse program in DQD acquisition mode was used with: NS = 32; TD = 2048 (F2), 512 (F1); SW = 15.0191 ppm (F2), 149.9819 ppm (F1); O2 (F2) = 2000.65 Hz, O1 (F1) = 7545.96 Hz; D1 = 2 s; CNST2 (^1^J(C-H) = 145; acquisition time F2 channel = 85.1968 ms, F1 channel = 8.4818 ms; snf pulse length of the 90°. High power pulse P1 was optimised for each sample.

NMR data were processed with MestreNova (Version 8.1.1, Mestrelab Research, Santiago de Compostela, Spain) by using a 60°-shifted square sine-bell apodisation window; after Fourier transformation and phase correction, a baseline correction was applied in both dimensions. Spectra were referenced to the residual signals of DMSO-*d*_6_ (2.49 ppm for ^1^H and 39.5 ppm for ^13^C spectra).

### 2.5. FT-IR Analysis

Fourier-Transform Infrared (FT-IR) spectra were measured on a Perkin Elmer Spectrum 100 FTIR spectrometer (Waltham, MA, USA) operated with Spectrum software (Version 6.3.5). The spectra were acquired in the form of potassium bromide pellets as the average of 32 scans between 450 and 4000 cm^−1^ with a resolution of 4 cm^−1^.

### 2.6. Gel Permeation Chromatographic Analyses

For gel permeation chromatography (GPC), approximately 2–3 mg of lignin were dissolved in HPLC-grade dimethyl sulfoxide (DMSO) (Chromasolv^®^, Sigma-Aldrich/Merck KGaA, Darmstadt, Germany) containing 0.1% (*m*/*v*) lithium chloride (LiCl). A Shimadzu instrument (Kyoto, Japan) was used consisting of a controller unit (CBM-20A), a pumping unit (LC 20AT), a degasser (DGU-20A3), a column oven (CTO-20AC), a diode array detector (SPD-M20A), and a refractive index detector (RID-10A), and controlled by Shimadzu LabSolutions (Version 5.42 SP3). For separation, three analytical GPC columns (each 7.5 × 30 mm) in series were used in series: Agilent (Santa Clara, CA, USA) PLgel 5 µm 10000 Å, followed by Agilent PLgel 5 µm 1000 Å, followed by an Agilent PLgel 5 µm 500 Å. HPLC-grade DMSO containing 0.1% (*m*/*v*) LiCl was used as eluent (0.75 mL min^−1^ for 70 min at 70 °C column temperature). Calibration was performed with polystyrene sulfonate standards (Sigma Aldrich/Merck KGaA Darmstadt, Germany) MW range 0.43–2.60 × 10^6^ g mol^−1^) in acid form, and lower calibration limits were verified by the use of monomeric and dimeric lignin models. Final analyses of each sample was performed using the intensities of the UV signal at λ = 280 nm employing a tailor-made MS Excel-based table calculation, in which the number average molecular weight (Mn) and the weight average molecular weight (*M*_w_) were calculated based on the measured absorption (in a.u.) at a given time (min) after corrections for baseline shift and drift as described before [37]. Analyses were run in duplicate.

### 2.7. Differential Scanning Calorimetry

Differential scanning calorimetry (DSC) was performed using a Mettler Toledo DSC/TGA 1 Calorimeter (Columbus, OH, USA) or a Mettler Toledo DSC 820 Calorimeter (Columbus, OH, USA). Typical sample amounts of around 2–4 mg were exactly weighed in 40 µL aluminium pans, which were closed with a lid that was centrally punctured to prevent pressure build-up. The following optimised temperature sequence was applied on all samples under a Nitrogen atmosphere (50 mL min^−1^): 25 °C to 105 °C to 25 °C to 400 °C at a rate of 10 °C/min. Analysis was performed using Mettler Toledo Star1 software (Columbus, OH, USA). Experiments were run in duplicate or triplicate if not stated otherwise.

## 3. Results and Discussion

### 3.1. **WS-OSL** Fractionation

**Figure 2:**
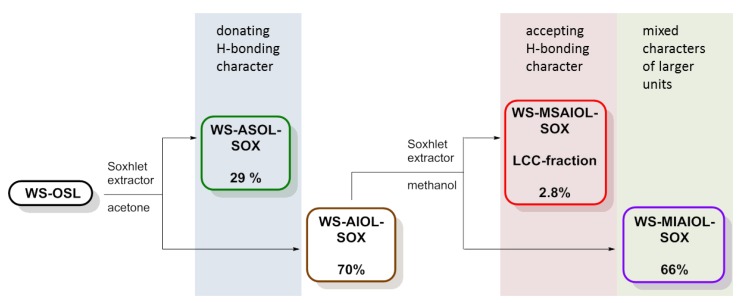
Flow of Soxhlet-based **WS-OSL** fractionation, leading to, among others, the LCC-fraction as the methanol soluble part of the acetone insoluble fraction (**WS-MSAIOL-SOX**).

In a straight forward fashion, **WS-OSL** was fractionated using a Soxhlet-extractor (Scheme 1): fractionation started with exhaustive extraction of powdery **WS-OSL** with dry acetone. Removal of solvent and drying of the solid residues at 40 °C in a vacuum oven furnished 29% of Soxhlet-derived acetone-soluble wheat straw organosolv lignin, i.e., **WS-ASOL-SOX**, and 70% of Soxhlet-derived acetone-insoluble wheat straw organosolv lignin, i.e., **WS-AIOL-SOX**. These results correspond to earlier findings in our group regarding the fractional precipitation of this **WS-OSL** [9]. In search for the more H-bonding-accepting parts of the **WS-AIOL-SOX**, this fraction was extracted with methanol. Novel fractions of Soxhlet-derived methanol-soluble acetone-insoluble and methanol-insoluble acetone-insoluble wheat straw organosolv lignin, **WS-MSAIOL-SOX** and **WS-MIAIOL-SOX**, respectively, were obtained in 2.8% and 66% yield, respectively (Figure 2 and Table 1).

Obtained fractions were analysed for molecular mass and structural characteristics using gel permeation chromatography, FT-IR spectroscopy, quantitative ^31^P NMR spectroscopy and semi-quantitative ^1^H–^13^C HSQC measurements. Molecular mass key figures are summarised in Table 1.

While acetobromination was successfully used in the past for **WS-OSL** analysis by our group [9,37], this sample preparation was banned in this study to avoid a partial destruction of the structure during sample preparation [38]. Instead, dimethyl sulfoxide (DMSO) was used as solvent for GPC analyses, since it could dissolve all samples generated in this study. To prevent aggregation of molecules by excessive electronic interactions between aromatic moieties and hydroxyl–π interactions, lithium chloride was added to the DMSO used for both sample preparation and chromatographic analyses. As expected in light of the differences in GPC analyses, molecular weight information generated in this study divert in terms of absolute numbers from previously reported mean average molecular weights (*M*_n_). This difference can be explained not only by the previously discussed changes in structure and aggregation behaviour introduced by the chemical derivatisation with acetyl bromide [38], but also with a change in aggregation behaviour in absolute terms in a polar solvent such as DMSO. Nevertheless, a comparison of the data obtained for **WS-ASOL-SOX** and **WS-AIOL-SOX** in the DMSO-based GPC-analyses with those obtained for the very same fractions in the optimised THF-based set-up indicates the comparability in relative terms and trends.

**Figure 3:**
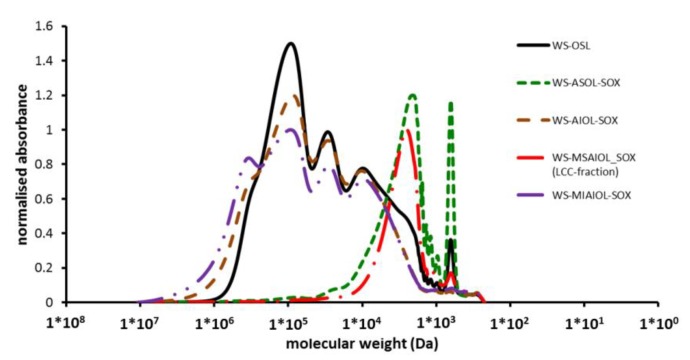
GPC-analyses of **WS-OSL** and Soxhlet-derived fractions thereof.

As expected, and graphically shown in Figure 3, **WS-ASOL-SOX** and **WS-AIOL-SOX** differ significantly in their molecular mass characteristics, with acetone insoluble **WS**-**AIOL-SOX** exhibiting roughly double the molecular weight.

Most interestingly, extracting the **WS AIOL-SOX** fraction with methanol yielded only a small amount (2.8%) of a methanol soluble fraction, **WS-MSAIOL-SOX**, that exhibited a molecular weight just slightly higher than that of the acetone soluble fraction **WS-ASOL-SOX**, i.e., 1900 Da vs. 1800 Da, respectively (Table 1 and Figure 3). Given that a Soxhlet-extraction represents an exhaustive procedure, this initial analysis hints at the fact that **WS-OSL** contains oligomers of relatively low molecular weight that exhibit H-bonding characteristics different from those of originally generated **WS-ASOL-SOX**. While the former is expected to be moderately polar and represent rather H-bond-donor characteristics, **WS-MSAIOL-SOX** should be significantly more polar and display both H-bond donating and accepting capabilities. It is worth mentioning that, using a “standard” solvent-based fractionation approach in which the generation of a methanol-soluble fraction would be followed by an acetone fractionation (e.g., [14]), such high-polarity-low-weight fractions remain in disguise, being eventually just part of the normally in abundance obtained “standard” methanol fraction.

### 3.2. Structural Features of **WS-OSL** Fractions

**Figure 4:**
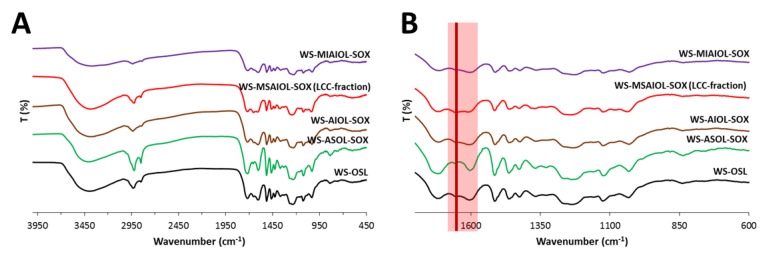
FT-IR spectra of **WS-OSL** and fractions derived thereof: full spectra (**A**); and fingerprint region (**B**).

To get a first hint at eventual very characteristic structural differences between the four fractions, FT-IR spectra were acquired. Generally, the spectra (Figure 4) display the bands expected for a wheat straw-derived organosolv lignin: hydroxyl groups centred around 3390 cm^−1^ are dominant; C–H stretching vibrations cause characteristic band patterns around 2935 and 2850 cm^−1^. Unconjugated C=O carbonyl groups cause a strong band at 1718 cm^−1^. A weak band identifiable at 1654 cm^−1^ can be explained by the presence of C=O stretching vibration in conjugated *para*-substituted aryl ketones. The strong band at 1603 cm^−1^ belongs together with the bands at 1514 cm^−1^ to the pattern of bands of the vibrating aromatic backbone, while peaks connected to asymmetric C–H deformation and stretching of aromatic rings and asymmetric C–H deformation of aromatic methoxy groups are found at 1463 and 1425 cm^−1^, respectively. Symmetric C–H deformation of the methoxy-groups of S-type and G-type aromatics are well reflected by the band identifiable at 1368 cm^−1^. The band at 1330 cm^−1^ indicates aryl breathing with C–O stretching in G-type and S-type aromatics, while the band at 1253 cm^−1^ stands for aryl breathing with C=O stretching. The esters present in the peripheries, i.e., the ferulates, coumarates and process-induced acetates, together with the alkyl-aryl ethers in the backbone cause a strong decrease of transmission in a range of approximately 75 cm^−1^ around the centre of 1250 cm^−1^ due to the stretching of their C–O motifs. Within the fingerprint region, distinct bands representing aromatic C–H in plane deformation are found at 1158 cm^−1^, 1125 cm^−1^ and around 1035 cm^−1^. Out of plane vibration modes of C–H bonds of the various types of aromatics would explain bands at varying intensities at, e.g., in the case of parent **WS OSL**, 882, 836 and 757 cm^−1^, respectively.

Only the methanol soluble part of the acetone-insoluble fraction, **WS-MSAIOL-SOX**, shows a noteworthy and interesting difference in the IR spectra: the band around 1650 cm^−1^ shows a significant increase in intensity relative to the “standard” **WS-OSL** bands around, especially in direct comparison with the band around at 1600 cm^−1^. This band around 1650 cm^−1^ is generated especially by α-β-unsaturated carbonyl groups, carbonyl groups involved in strong intramolecular hydrogen bonding, aromatic aldehydes, aryl ketones and enol ethers as well as quinone structures. Such structures would be expected to be present, albeit in varying concentrations in all **WS-OSL** fractions generated in the presented fractionation approach based on H-bonding affinities. The clear overexpression of this motif, in light of the process yielding this fraction, points to an eventual enrichment of structural features, i.e., α-β-unsaturated carbonyl groups, often claimed to be indicative of lignin carbohydrate complexes as impurities in **WS-OSL** [13,39].

**Figure 5:**
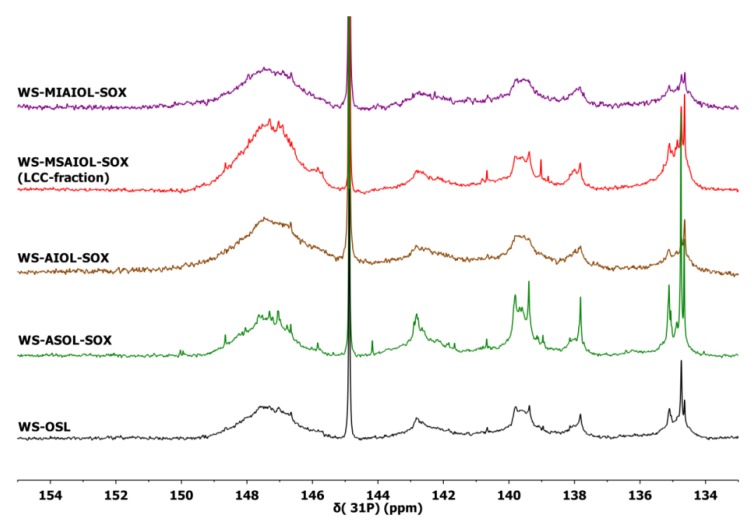
Quantitative ^31^P NMR spectra obtained for **WS-OSL** and fractions derived thereof, after phosphitylation using 2-chloro-4,4,5,5-tetramethyl-1,3,2-dioxophospholan.

More detailed structural insight was sought by subsequent analyses of the fractions by quantitative ^31^P NMR spectroscopy, allowing for identifying further differences in end groups and hydroxylated interunit bondings. Data are summarised in Table 2. Figure 5 gives a comparison of ^31^P NMR spectra.

Comparative analysis of the ^31^P NMR indicate an expected trend in the phenolic groups contents of the acetone soluble and insoluble fractions [9]. The **WS-MIAIOL-SOX** fraction exhibits the overall lowest hydroxyl group content with only 3.01 mmol/g (Table 2, entry 5). More specifically, the analysis of **WS-MIAIOL-SOX** reveals very similar data for aliphatic OH-groups, mono- and un-substituted phenolic OH-groups and carboxylic acid groups in comparison to parental **WS-AIOL-SOX**, but an interesting depletion in phenolic OH, especially in syringyl units (Table 2, entry 5). This can be interpreted as a net reduction of condensed structures, or a depletion in S-type phenolics. As found before, the acetone-soluble fraction is generally enriched in phenolic end-groups, while the acetone insoluble fraction shows a lower phenolic group content. 

Undoubtedly most interesting is the low molecular weight oligomeric methanol-soluble fraction **WS-MSAIOL-SOX**. Along with a similarity to the parent **WS-OSL** in the relative distribution of aromatic OH-groups, it exhibits a significantly increased content of aliphatic OH-groups and an elevated acid content (Table 2, entry 4). These findings suggest that this **WS-MSAIOL-SOX** fraction is eventually *enriched* in structures that are considered typical for being part of wheat straw organosolv lignins as reminiscence of the isolation process: residues of polycarbohydrates linked to lignin fragments via ferulates and coumarates [13,31,32,40,41]. In this, the ^31^P NMR-based finding corresponds to the FT-IR results, indicating an elevated presence of ferulates and coumarates that act as link between lignin and carbohydrates and thus being the key part in postulated lignin carbohydrate complexes (LCCs).

**Figure 6:**
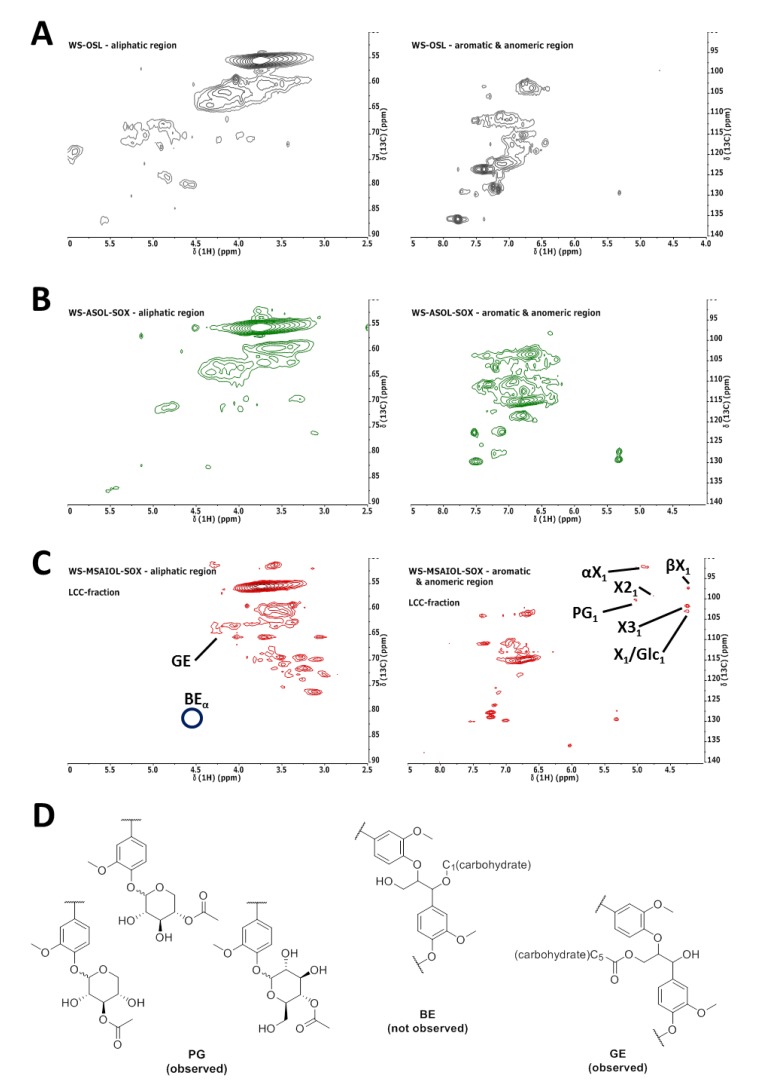
^1^H–^13^C HSQC spectra obtained for: (**A**) **WS-OSL** [9]; (**B**) **WS-ASOL-SOX**; and (**C**) **WS-MSAIOL-SOX** (PG_1_, phenyl glycoside (linkages); αX_1_, (1→4)-α-d-xylopyranoside; βX_1_, (1→4)-β-d-xylopyranoside; X3_1_, 3-*O*-acetyl-β-d-xylopyranoside; X_1_/Glc_1_, β-d-xylopyranoside/β-d-glucopyranoside). (**D**) Structural representation of postulated LCC motifs (**PG**, phenyl glycoside; **BE**, benzyl ether; **GE**, γ-ester). Assignments in (**C**) are based on earlier reports [13,25,26]. Circles in (**C**) indicate positions of cross peaks that would be indicative of benzyl ether-type LCC-structures, indicated as “not observed” in (**D**).

Further spectroscopic proof for the hypothesis that **WS-AIOL-SOX** represents LCCs was obtained by comparative ^1^H–^13^C HSQC analysis of parent **WS-OSL** [9] and soluble fractions **WS-ASOL-SOX** and **WS-MSAIOL-SOX**. Spectra of fractions were acquired using fixed analysis conditions in terms of concentrations of samples and acquisition parameters, working with non-acetylated samples. While acetylation was used before in the acquisition of HSQC spectra of wheat straw samples to render them more soluble at higher concentrations, this was omitted in this study to not introduce any undesired structural changes potential induced by the conditions prior to analysis. More specifically, a transesterification of potentially ester-linked carbohydrates, as well as peracetylation of the sugars, was sought to be avoided. Obtained HSQC-spectra are shown in Figure 6.

HSQC-spectra obtained for the soluble fractions (Figure 6A–C) correspond the findings delineated so far on the basis of the other spectroscopic analysis techniques: **WS-ASOL-SOX** shows similarities to the parent **WS-OSL** by and large, exhibiting characteristic cross-peaks for relevant lignin interunit bondings, as discussed before [9]. Distinct changes can be seen in the aromatic region. The cross-peaks typical for S-type aromatic units are more intense in the soluble fraction, a trend indicated also by ^31^P NMR end group analysis. The region between 120 and 140 ppm is differently populated, indicating ferulates, coumarates and *para*-hydroxy-benzoate contents were changed upon extraction. This indicates that especially those ferulates and coumarates were removed that are still bound to carbohydrate residues. 

Methanol-soluble subfraction **WS-MSAIOL-SOX**, on the other hand, results in a spectrum clearly assigned to LCCs. Cross-peaks indicative of various carbon atoms pertinent to xylopyranosides are detectable, as detailed in Figure 6C. Analysis of the cross-peaks obtained for this fraction, confirmed additionally by running spectra after doubling of the sample concentration, hints at the presence of phenyl glycosides and potentially γ-esters (**GE**) as LCC motifs. Whereas the signal at 98.4/4.90 is interpreted as typical for phenyl glycosides (**PG**), the cross-peak at around 63.2/4.40 reveals an esterified gamma-position in β-*O*-4’ subunits, potentially linked to carbohydrates [13,25,26].

Other cross-peaks eventually indicative of benzyl ethers as indicated in Figure 6D are not detectable. Given that a sample preparation potentially leading to an ester cleavage upon transesterification has been avoided, this finding potentially suggests that the organosolv process used for the isolation of the **WS-OSL** under study [27] successfully cleaved benzyl ethers while not fully removing phenyl glycosides.

Since HSQC analyses show that **WS-MSAIOL-SOX** represents an “LCC-fraction”, **WS-MIAIOL-SOX** should represent a fraction of “purified” lignin of higher molecular weight. While this could not be confirmed by HSQC analysis due to a decreased solubility of this sample under the chosen conditions omitting an acetylation, it is in line with the ^31^P NMR results discussed above. Obviously, this study does not manage to indicate whether the detectable bonds between the carbohydrate moieties and the lignin oligomers are leftovers from an original linking between lignin and hemicellulose in the biomass, or artefacts of the isolation process.

### 3.3. Thermal Properties of Soxhlet-Derived **WS-OSL** Fractions

It can be expected at this point that the removal of 4% of a LCC-fraction should have an effect on the thermal properties of the isolated lignins. The glass transition temperatures (*T*_g_) obtained for the various fractions by differential scanning calorimetry actually supports this expectation, as the results listed in Table 3 indicate.

**Figure 7:**
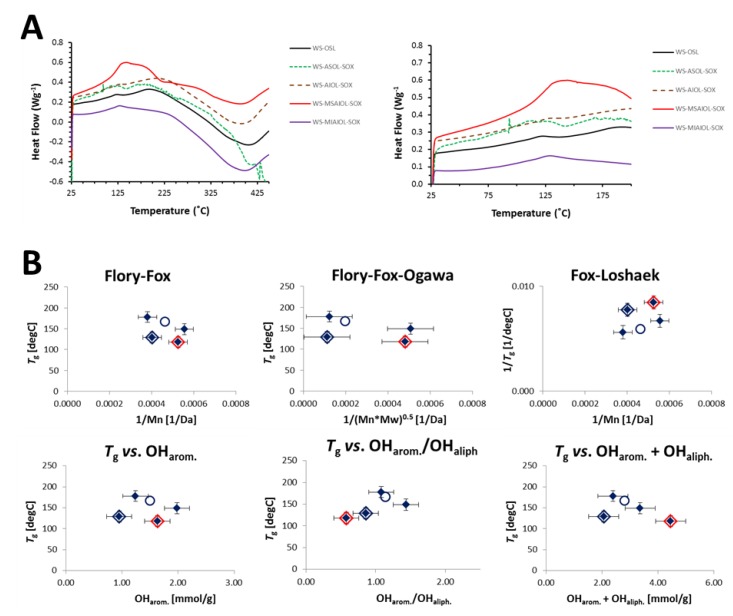
(**A**) Heat flow curves obtained for **WS-OSL** and fractions derived thereof. (**B**) Correlation of glass transition temperatures with molecular weight characteristics according to Flory–Fox, Flory–Fox–Ogawa and Fox–Loshaek theories, respectively, as well as directly with the mean average molecular weight (Mn), the total amount of phenolic OH-groups and the ratio between aromatic and aliphatic OH-groups. **WS-OSL** is represented by the hollow circle, **WS-MSAIOL-SOX** is additionally evidenced by a red rhomb, and **WS-MIAIOL-SOX** by a blue rhomb.

Removal of the “LCC-fraction” **WS-MSAIOL-SOX** from **WS-AIOL-SOX** results in a drastic decline of the glass transition temperature of the resulting **WS-MIAIOL-SOX**: this highest MW-fraction has the lowest *T*_g_ of all “lignin fractions”, i.e., all fractions except the LCC-fraction **WS-MSAIOL-SOX**. In light of the high molecular weight of this fraction, this observed drastic decline shows the overall higher importance of the phenolic OH-group content for the glass transition temperature. **WS-MIAIOL-SOX** has the lowest relative phenolic OH-group concentration per mass of all lignin fractions; generally, it has the lowest OH-group content, as discussed before. A direct comparison of the heat flow curves generated for the various fractions (Figure 7A) reveals an overall similarity between the acetone-insoluble fraction **WS-AIOL-SOX**, its additionally methanol-insoluble daughter fraction **WS-MIAIOL-SOX** and the parent **WS-OSL**. **WS-ASOL-SOX** is the only fraction that shows significant decomposition even at relatively low temperatures of around 400–450 °C. LCC fraction **WS-MSAIOL-SOX** is very different with respect to the lignin fractions, exhibiting the highest net heat uptake, and the most pronounced glass transition step.

It has been shown before that the fractions obtained by fractional precipitation from an acetone–hexane system correlate rather well with molecular weight characteristics according to standard correlations such as both the classical Flory–Fox theory [42], which was shown to perform more reliably for larger, rather narrowly polydispersed polymers, and the Okawa variation of the Flory–Fox equation that was developed for improved description of more polydispersed polymeric samples [43]. For both correlations, acetone-soluble hexane-precipitated fractions exhibiting neither perfect nor extremely large polydispersities yielded linear trends with acceptable coefficients of determination [9]. For the Fox–Loshaek variation [44] accounting more explicitly for an eventual crosslinking of the polymer, a less good correlation was found. Such correlation is not found for the fractions generated in this study (Figure 7B).

Rough correlations were only found for **WS-OSL** and the two initial acetone fractions **WS-ASOL-SOX** and **WS-AIOL-SOX**. Rough correlations were also found when plotting glass transition temperatures against total OH-group content, i.e., the concentration of aliphatic and aromatic OH-groups, and their ratios.

Accumulated evidence hence suggests that the various fractions do not exhibit a homogeneous family in any respect; as such, the thermal data correspond to the spectroscopy-based structural insights. The discussed fractionation protocol employing two polar solvents with different H-bonding characteristics rather yields a split of different types of lignin fractions from **WS-OSL**. While the LCC fraction, i.e., **WS-MSAIOL-SOX**, can be seen unique in the range of fractions, the mismatch of **WS-MIAIOL-SOX** in light of its spectroscopic similarity to **WS-AIOL-SOX** makes it necessary to gain further structural insight. Different analysis techniques than NMR would be necessary, however, since the overall insolubility under the standard conditions impedes knowledge generation here.

## 4. Conclusions

The presented fractionation approach using simply acetone as aprotic polar solvent and methanol as protic polar solvent in a Soxhlet-base exhaustive extraction succeeded in separating wheat straw fractions with distinct structural and molecular weight features. Most interestingly, the fraction obtained upon methanol extraction of acetone-insoluble material yielded a fraction of isolated lignin carbohydrate complexes (LCCs) that have been removed from the bulk lignin. The proposed protocol can thus be used with the double purpose of LCC isolation and **WS-OSL** purification from residual polyphenol-linked carbohydrate impurities. The obtained, “purified” lignin fractions themselves show spectroscopically and thermally distinct features, pointing at a structural heterogeneity of the **WS-OSL** that has yet been potentially overlooked.

## Figures and Tables

**Table 1:** Yields and molecular mass key characteristics of **WS-OSL** and Soxhlet-derived fractions.

Entry	Sample	Yield (%)	Mn (Da)	Mw (Da)	PDI ^a^
1	**WS-OSL**	---	2200	12,100	5.6
2	**WS-ASOL-SOX**	29	1800	2200	1.2
3	**WS-AIOL-SOX**	70	2600	25,200	9.6
4	**WS-MSAIOL-SOX (LCC-fraction)**	2.8	1900	2300	1.2
5	**WS-MIAIOL-SOX**	66	2500	32,000	>10

^a^ Polydispersity index.

**Table 2:** OH-group contents obtained for **WS OSL** and solubility-based fractions thereof after phosphitylation using 2-chloro-4,4,5,5-tetramethyl-1,3,2-dioxaphospholane.

Entry	Sample	Aliphatic OH (mmol/g)	Aromatic OH (mmol/g)			Acidic OH (mmol/g)	Total OH (mmol/g)
			Siringyl	Guaiacyl	*p*-hydroxy		
1	**WS-OSL**	1.31	0.63	0.59	0.27	1.48	4.28
2	**WS-ASOL-SOX**	1.38	0.85	0.82	0.31	1.99	5.35
3	**WS-AIOL-SOX**	1.15	0.64	0.39	0.21	1.24	3.63
4	**WS-MSAIOL-SOX (LCC-fraction)**	2.82	0.66	0.67	0.30	1.63	6.08
5	**WS-MIAIOL-SOX**	1.11	0.37	0.38	0.20	0.95	3.01

**Table 3:** Glass transition temperatures of **WS-OSL** and fractions delineated thereof.

Sample	OSL	ASOL-SOX	AIOL-SOX	MSAIOL-SOX (LCC-fraction)	MIAIOL-SOX
*T*_g_ (°C) ^a^	168	149	178	118	129

^a,^ As determined by DSC analysis. In case of multiple detectable glass transitions, the lowest one is listed here.
